# Risk Factors for Symptomatic Pericardial Effusions Posthematopoietic Stem Cell Transplant

**DOI:** 10.1155/2023/7455756

**Published:** 2023-02-08

**Authors:** Kelly Lyons, Niti Dham, Bryanna Schwartz, Blachy J. Dávila Saldaña

**Affiliations:** ^1^Children's National Hospital, Division of Bone Marrow Transplant, Washington, Columbia, USA; ^2^Children's National Hospital, Division of Cardiology, Washington, Columbia, USA; ^3^Department of Pediatrics, George Washington University School of Medicine and Health Sciences, Washington, Columbia, USA

## Abstract

**Background:**

Pericardial effusions are a known complication posthematopoietic stem cell transplant (HSCT), causing significant morbidity. We aimed to evaluate the risk factors associated with the development of high-grade effusions requiring interventions. *Procedure*. A retrospective chart review of all HSCT patients over a period of 7 years (2013–2019) in a single institution in the Northeastern United States is conducted. All patients who developed an effusion requiring intervention were included. Patient's clinical characteristics were compared with all others transplanted during the same time period. Echocardiogram findings of the affected patients were compared to a case-control cohort of unaffected patients with similar age and diagnosis. Chi-square and paired *t*-tests were utilized to ascertain statistical differences between the groups.

**Results:**

A total of 15 patients out of 201 (7.5%) transplanted at our institution developed a moderate or large pericardial effusion requiring pericardiocentesis or a pericardial window. Of this cohort, 13 (87%) underwent a myeloablative preparative regimen, 13 (87%) had cyclophosphamide as part of their regimen, 13 (87%) had recent treatment for viral reactivation, 6 (40%) had an underlying hemoglobinopathy diagnosis, and only 4 (27%) had an active diagnosis of GVHD. A myeloablative preparative regimen had a higher rate of effusion requiring intervention, although it was not statistically significant, and concurrent GVHD was not predictive of effusion development. However, exposure to cyclophosphamide, recent treatment for viral reactivation, and a diagnosis of transplant-associated thrombotic microangiopathy (Ta-TMA) were highly associated with effusions. The latter was associated with increased mortality. The duration of pericardial effusion correlated with the pretransplant echocardiogram left ventricle end diastolic diameter z-score and apical 4-chamber left ventricular peak average strain measurement.

**Conclusions:**

Potential risk factors for pericardial effusions post-HSCT include a diagnosis of Ta-TMA, active viral infection, exposure to cyclophosphamide, and a higher left ventricle end diastolic diameter *z*-score. This information may help guide management for these patients, including identifying high-risk subjects, determining the frequency of echocardiograms, and determining specific echocardiogram measures to follow over time.

## 1. Introduction

Hematopoietic stem cell transplant (HSCT) is a curative treatment for many malignant and nonmalignant conditions, including but not limited to cancer, hemoglobinopathies, and primary immunodeficiency syndromes. Pericardial effusions (PEF) are emerging as a more commonly reported condition in the peritransplant period in pediatric patients undergoing HSCT. Most pediatric PEF have been described in patients with a concurrent inflammatory process, so it stands to reason that HSCT patients would be at risk for the development of this complication [1]. Medical management can be attempted, but a significant number will require invasive drainage via pericardiocentesis when concerns for tamponade are present. Given the multiple comorbidities associated with HSCT, PEF requiring pericardiocentesis can be a high morbidity procedure [2]. Prior studies have shown PEF to be associated with worse overall survival after transplant and, in some studies, a significant risk factor for posttransplant mortality [2, 3]. Therefore, addressing inflammatory triggers to prevent such procedures and identifying patients at higher risk for developing more severe PEF would have a high yield. The inciting factors associated with PEF development in HSCT patients, however, are not well described. In addition, to our knowledge, there is limited literature describing echocardiogram findings that predict the development and severity of PEF after HSCT.

Rhodes et al. [4] had one of the first and largest reviews of pericardial effusions in pediatric stem cell transplant; they described an incidence rate of 4.4% of all patients over a 10-year study period, all with graft versus host disease. Almost a decade later, Aldoss et al. [5] described an incidence of 19% of allogeneic transplant recipients developing PEF. Other described associations include viral infections, transplant-associated thrombotic microangiopathy (TA-TMA), [6] the conditioning regimen utilized, and subsequent engraftment. Specifically, myeloablative preparative regimens, inclusion of total body irradiation, and delayed neutrophil engraftment have all been shown to correlate with PEF [5]. Relationships between underlying comorbid cardiac conditions and PEF in HSCT patients are not as frequently described.

An echocardiogram is a well-validated technique for diagnosing pericardial effusions, as relying on clinical findings alone can be difficult [1]. Echocardiograms can assess the degree of the PEF as well as a general sense of cardiac systolic function. It does have its limitations in assessing cardiac diastolic function. Cardiac strain is a more novel echocardiogram measurement that has shown to be volume independent and an even better marker of early cardiac toxicity in cancer survivors [7, 8].

We sought to show associated risk factors for pediatric HSCT patients that developed PEF requiring invasive treatments and to explore the predictive value of pretransplant echocardiograms obtained at diagnosis and resolution.

## 2. Methods

### 2.1. Patient Selection

We performed a retrospective chart review of all patients who received an allogeneic hematopoietic stem cell transplant at Children's National Hospital during the study period of January 2013 to December 2019. This study was approved by the IRB, and a waiver of informed consent was obtained during IRB approval.

If a patient developed a PEF of moderate or larger size as evaluated on a transthoracic echocardiogram, all pertinent clinical documentation, diagnostic imaging, medication records, laboratory results, and pathology reports were reviewed. Patients were considered symptomatic if they had otherwise unexplained tachycardia, tachypnea, or hypotension, or if there were signs of atrial collapse on imaging. No patients were excluded. All patients included in the review were subject to standard pretransplant infectious screening as per institutional standards, which included (but was not limited to) CMV, EBV, HSV, and toxoplasma serologies. In addition, all patients underwent weekly PCR serum screening for CMV, adenovirus, and EBV starting at initiation of the preparative regimen until absolute CD4 counts were sustained at over 200 k/mcl. Patients with a history of significant structural heart disease or cardiac surgery were excluded.

Echocardiogram data for patients developing PEF were then compared to a case-control cohort. All patients included in the control cohort underwent HSCT during the study period but did not develop a pericardial effusion. Subjects were matched by diagnosis, age at BMT, donor source, and preparative regimen.

### 2.2. Echocardiogram Review

All subjects had at least three echocardiograms reviewed during the study period: pretransplant (baseline), at the time of PEF diagnosis, and at the resolution of PEF. Of note, for patients being transplanted for malignancy, the baseline echocardiograms used were the ones done prior to transplant and not necessarily prior to their pre-HSCT treatment. Patients had multiple echocardiograms for follow-up related to medical care, but PEF was considered resolved when it was read as trivial with no further record of recurrence or progression. For subjects in the case-control cohort, the pre-HSCT echocardiogram was used.

All echocardiogram measurements, including the size of effusions, were reviewed for accuracy by an experienced pediatric cardiologist if included in our study. From each echocardiogram, measures for systolic function (ejection fraction and shortening fraction), diastolic function (mitral valve *E*/*A* and mitral valve tissue doppler imaging), left ventricular size (left ventricular end diastolic diameter), and left ventricular strain were collected. Strain measurements were performed on the PEF subjects and controls. Patients without adequate quality echocardiogram images for strain analysis were excluded from this additional analysis.

### 2.3. Statistical Analysis

Pearson's chi-squared test was used to determine whether a statistically significant difference was present between the expected and observed frequencies of patients with pericardial effusions as compared to all transplanted patients in one or more of these categories: presence of graft versus host disease, the preparative regimen used (myeloablative vs. reduced intensity), and presence of Ta-TMA. A paired *t*-test was performed to compare echocardiographic measures of systolic function, diastolic function, LV size, and LV strain between the PEF and control groups. A two-tail Pearson correlation was performed to assess the correlation between duration of effusion and body surface area, hemoglobin, and pretransplant echocardiogram variables.

## 3. Results

A total of 50 patients out of 201 transplanted during the study period developed a PEF, with 70% self-resolving and only 15 becoming symptomatic (7.5%). All symptomatic patients required invasive intervention including pericardiocentesis (*n* = 15) and/or a pericardial window (*n* = 1). Ages ranged from 7 months old to 20 years old, with a mean and median of 7 years of age. Most pericardial effusions occurred before day 100 (80% of patients), although the date of diagnosis ranged from *D* + 32 to *D* + 915, with median time from transplant being 53 days. None of these cases were associated with rejection of the graft. Only one patient was diagnosed more than 5 months posttransplant (day + 915); this was in the setting of acute influenza A infection in a patient with bronchiolitis obliterans. Immunosuppression was calcineurin inhibitor (CNI) based on all patients with PEF. One patient was on no immunosuppression at the time of diagnosis because of the extent of her malignancy; the 14 other patients were on a CNI. Four patients were on multiple immunosuppressive agents, including the addition of steroids (*n* = 4), mycophenolate mofetil (*n* = 2), extracorporeal photopheresis (ECP) (*n* = 1), or sirolimus (*n* = 1). Subjects are described in more detail in Table 1. Medical interventions varied and included but were not limited to steroids, NSAIDS, diuretics, and rituximab.

Of the 201 patients, 132 (66%) received a myeloablative preparative, 92 (46%) experienced any form of acute GVHD, and 11 (5%) had a diagnosis of ta-TMA. 130 (65%) of the patients in the study period were transplanted for a nonmalignant condition, whereas the remaining 71 (35%) were transplanted for a malignant condition.

In the 15 patients who experienced a pericardial effusion requiring intervention, 13 (87%) received a myeloablative preparative regimen. We further noted a predominance of cyclophosphamide-containing regimens: 13 of the 15 (87%) patients who experienced PEF received cyclophosphamide. Looking at all transplanted patients, 13 of the 123 (11%) patients receiving cyclophosphamide developed effusion, compared to 2 of the 78 (2.6%) who did not (*p*=0.04).

Of those with a significant effusion, 8 (53%) experienced any form of acute GVHD, and 3 (20%) had a diagnosis of ta-TMA. There was a slight male predilection (66% of cases). Five (33%) had malignant diagnosis, with 67% having nonmalignant disorders. While this is comparable to the whole cohort transplanted over the study period, most of these patients (*n* = 5) had been transplanted for hemoglobinopathy. Only 4 (27%) had active GvHD at the time of PEF diagnosis. More patients undergoing myeloablative transplants developed effusions, though this difference was not statistically significant (*p*=0.08). Similarly, there was no significance in the correlation between rates of GVHD in patients with an effusion requiring an intervention and those who did not (*p*=1). A diagnosis of Ta-TMA, however, was found to be a statistically significant risk factor for developing PEF, requiring medical and surgical intervention (*p*=0.01). These comparisons are shown in more detail in Table 2.

Thirteen of the 15 patients (87%) had viral infections and were receiving or had received antiviral treatment within 2 weeks of diagnosis. While several patients had more than one active infection, CMV was the most common diagnosis (*n* = 8), followed by influenza (*n* = 5) and Epstein Barr Virus (EBV) [*n* = 5]. Other diagnoses included metapneumovirus (*n* = 3), adenovirus (*n* = 2), parainfluenza (*n* = 1), and parvovirus (*n* = 1). The two patients without a viral diagnosis had events related to their diagnosis of malignancy which were presumed to be related to the effusion (mediastinal mass = 1 and relapsed leukemia = 1). All drained effusions were sent for analysis of the pericardial fluid, which included cytology, infectious, and inflammatory analysis. Fluid analysis was negative in 80% of cases. Positive cases, all identified by PCR, included CMV (*n* = 1), parvovirus (*n* = 1), and EBV (*n* = 1). Four cases (27%) required a second intervention, be it repeat drainage or the placement of a pericardial window. Three of these four cases (75%) were those with infections identified in the pericardial fluid.

While surgical interventions increased the length of hospital stays, there were no direct or severe complications associated with the procedures. Eleven patients (73%) had eventual full resolution of the PEF with a mean duration of effusion of 98 days. All these patients are alive and well. Four patients (27%) died after the development of PEF. All 3 patients who met the criteria for Ta-TMA and had pericardial effusion died. Causes of death include CMV pneumonitis, multiorgan failure from disseminated adenoviremia, and refractory GvHD. The fourth patient died of relapsed malignancy.

When comparing pretransplant echocardiogram variables of systolic function between patients requiring pericardiocentesis and controls, there was no significant difference between the ejection fraction (*p*=0.44), the shortening fraction (*p*=0.06), or the left ventricle end diastolic diameter *z*-score (*p*=0.19). The mean hemoglobin levels between groups were similar (*p*=0.38). There were no other statistically significant differences in measures of diastolic function between groups, including mitral valve *E* wave maximum velocity, mitral valve *E*/*A* ratio, and mitral valve tissue doppler. There was no difference in pre-BMT left ventricular peak average strain measurements between the subjects and controls (Table 3). The mean ejection fraction and fractional shortening were normal in the PEF group before effusion, at diagnosis of the effusion, and upon resolution of effusion, with no significant difference between these time periods (*p*=0.6 and *p*=0.7).

The duration of PEF correlated with pretransplant apical 4-chamber left ventricular peak average strain measurements (*r* = 0.68 and *p*=0.04) and the pretransplant left ventricular end diastolic diameter z-score (*r* = 0.52, *p*=0.07). There was no significant correlation found between duration of PEF and pretransplant ejection fraction, shortening fraction, hemoglobin level, mitral valve *E*/*A* ratio, or mitral valve tissue doppler imaging (Table 4).

## 4. Discussion

We report one of the largest single-institution reviews of pericardial effusions requiring medical and surgical intervention in a pediatric hematopoietic stem cell patient population. Our analysis also collected and analyzed data related to pretransplant cardiac function and function at the time of onset and resolution of the pericardial effusion.

Over a period of 6.5 years, we noted that 25% of our patients had any degree of pericardial effusion. Capillary leak is a well-described complication of HSCT, which likely accounts for this high number. However, most of these were self-resolving, and only 7.5% of our patients with pericardial effusions required drainage. We explored factors possibly associated with this development, including graft versus host disease, infections, the preparative regimen, disease, Ta-TMA, and echocardiogram data.

In our cohort, there is a trend for more intense myeloablative preparative regimens to be associated with PE, although this did not reach statistical significance. Given the difference in numbers, however, it is possible this would become significant with a bigger sample size. The use of cyclophosphamide was a statistically significant factor in the development of pericardial effusion. While cardiac necrosis is a rare but well-described side effect of cyclophosphamide, it is also considered to cause significant endothelial injury [9]. This likely underlies the association noted here with PEF development. Interestingly, graft versus host disease was not associated with PEF in our cohort. This is in contrast with multiple other previous studies. As these studies were conducted in decades past, it is likely most, if not all, of the transplants included in those studies underwent a myeloablative regimen. It is possible that the higher rate of reduced-intensity conditioning in our cohort may account for this difference.

An interesting observation is that most cases were in the setting of active infections, predominantly viral, with 9 patients having detectable viremia and/or being on viral treatment, plus an additional 4 patients having a recent active viral infection at the time of PEF diagnosis. One patient had an active fungal infection at PEF diagnosis. Furthermore, the presence of a viral infection in the pericardial fluid was the single factor present in the majority of PEF (*n* = 3) that required more than one intervention. This strongly argues that inflammation secondary to these infections is an important risk factor for PEF development. 80% of effusions occurred in the first 100 days, and all but one patient was still on immunosuppression, which is consistent with the hypothesis that endothelial injury posttransplant and poor immune function are relevant factors in the development of PEF. Data for viral reactivation were not captured for all the time frame of the study, which hinders our ability to analyze this as a risk factor for PEF. Previous research suggests viral reactivations occur in about 2/3 of transplant recipients, with 2/3 of these, or about 45% of all patients, requiring treatment for the reactivations [10]. This is substantially less than what was seen in our cohort (87%).

Lastly, Ta-TMA was found to be a high-risk factor for the development of PEF. Three patients in our cohort of PEF had Ta-TMA. This represents 20% of our cohort, and 27% of all patients with Ta-TMA were diagnosed within the study period. Ta-TMA has previously been described as a risk factor for the development of pericardial effusions in pediatric HSCT recipients [11]. Shockingly, all patients with a diagnosis of both Ta-TMA and PEF died, though none directly because of the PEF. This suggests PEF is a marker of severe Ta-TMA, and screening echocardiograms should be pursued in all patients with this diagnosis. A prior prospective single-center study also demonstrated an association of abnormal echocardiograms with Ta-TMA; however, in this study, the abnormality noted on the echocardiogram was associated with increased right ventricular pressure at day 7 after transplant [6]. Of note, over our study period from 2013 to 2019, there have been notable advances in the diagnosis of ta-TMA through increased laboratory surveillance for the syndrome. This will hopefully lead to more prompt diagnosis and, therefore, a lower mortality risk.

Overall, there was no statistically significant difference between the control and PEF groups for most systolic and diastolic function variables, as well as strain, on the pretransplant echocardiograms. A prior case-control study evaluating tissue doppler imaging before and 3 months after HSCT found subtle abnormalities in measures of diastolic function between the two groups [12]. However, our tissue doppler imaging measurements did not show a significant difference.

Pretransplant 4-chamber left ventricular peak average strain measurements and left ventricular end diastolic diameter *z*-score were both correlated with longer duration of PEF. The pretransplant ejection fraction and the shortening fraction did not correlate with duration of the pericardial effusion. To our knowledge, these findings have not previously been reported in the literature and suggest we should pay closer attention to the left ventricular size and strain measurements during pretransplant surveillance echocardiograms.

While still important in the pretransplant evaluation for cardiac toxicity, ejection fraction and shortening fraction were not found to add predictive value for the development of a PEF requiring an invasive intervention. The left ventricular ejection fraction has several limitations, including interobserver variability, and a change in ejection fraction is often a late finding of cardiotoxicity in patients after chemotherapy treatment [13, 14]. Recently, the literature has supported evaluation of myocardial deformation through measurement of left ventricular strain [13, 14], which was also noted in our study. Another study also noted that echocardiography may not identify all subclinical cardiac injuries and that biochemical abnormalities, including cardiac troponin and soluble suppressor of tumorigenicity, are often noted to be abnormal and may be important to follow in this population [15].

It is important to note that on the day of diagnosis, PEF were often incidentally found when either chest imaging or an echocardiogram was obtained for another purpose related to infection surveillance or vital sign changes. This would argue that there is a need for more frequent monitoring for the development of PEF in these patients. However, the frequency of this monitoring has not been well delineated [2, 3]. A prior single-center study implemented echocardiographic screening for all HSCT recipients admitted to the ICU with respiratory distress, hypoxia, shock, and complications related to ta-TMA and found abnormalities in 50% of patients (13% with pericardial effusions) [16]. However, many would argue that waiting to screen by echocardiogram until a patient is admitted in critical condition is too late. Developing a standardized algorithm for echocardiogram surveillance and PEF treatment based on the risk factors identified in this study may be an important next step in identifying PEF sooner and preventing the need for invasive interventions.

There are several limitations to our study, including its retrospective nature, which limited us to the quality and quantity of previous echocardiograms, as well as the documentation of other complications. However, our data suggest that patients receiving myeloablative preparative regimens, particularly when involving cyclophosphamide, those having viral reactivations, as well as all patients diagnosed with Ta-TMA should be prospectively screened for PEF, particularly in those known to have a higher left ventricle end diastolic diameter z-score prior to HSCT. These findings should be validated in multicenter, prospective studies.

## Figures and Tables

**Table 1:** Characteristics of patients developing pericardial effusions.

Factors	*N*	%
Overall	15	100
Sex		
Male	10	66.7
Female	5	33.3
Diagnosis		
Malignancy	5	33.3
Hemoglobinopathy	6	40
Immunodeficiency	4	26.7
Conditioning regimen		
Myeloablative	13	86.7
With cyclophosphamide	13	86.7
Reduced intensity	2	13.3
Hx CMV reactivation		
Yes	7	46.7
No	8	53.5
Active infection within 2 weeks of PEF diagnosis		
Yes	13	86.7
No	2	13.3
GVHD active at PEF dx		
Yes	4	26.7
No	11	73.3
PEF diagnosis and time from BMT		
Under day +100	12	80.0
Over day +100	3	20.0
Patients on immunosuppression at time of PEF diagnosis		
Yes	14	93%
No	1	7%

CMV, cytomegalovirus; PEF, pericardial effusion; GVHD, graft versus host disease.

**Table 2:** Predisposing risk factors for PEF development.

Characteristics	Overall cohort, *n* = 201	PEF, *n* = 15	*P* value
Preparative regimen			
RIC	69 (34%)	2 (13%)	
MAC	132 (66%)	13 (87%)	0.08
Underlying disease			
Malignant	71 (35%)	5 (33%)	
Nonmalignant	130 (65%)	10 (67%)	1
History of acute GVHD			
Yes	92 (46%)	8 (53%)	
No	109 (54%)	7 (47%)	1
History of TA-TMA			
Yes	11 (5%)	3 (20%)	0.01
No	190 (95%)	12 (80%)	

RIC, reduced intensity chemotherapy; MAC, myeloablative chemotherapy; GVHD, graft versus host disease; PEF, pericardial effusion; TA-TMA, transplant-associated thrombotic microangiopathy.

**Table 3:** Pretransplant comparison of cardiac markers.

	PCE subjects		Controls		
	*N*	Mean	*N*	Mean	*P* value
Number of days after BMT	15	121			
Duration of effusion (days)	15	98			
Hemoglobin (gm/dl)	12	9.3	15	9.9	0.38
Ejection fraction (%)	15	65	15	66	0.44
Shortening fraction (%)	15	37	15	39	0.06
LVIDd (cm; *z*-score)	13	0.9	15	0.4	0.18
MV *E* max vel (cm/sec)	13	107.1	15	110.7	0.49
MV *E*/*A*	13	1.6	15	2.1	0.21
Mitral *E*/TDI *E*′ (septal)	12	8.6	12	8.2	0.72
Mitral *E*/TDI *E*′ (lateral)	12	7.2	12	7.4	0.80
Apical 4 Ch LV peak avg	9	−18.07	14	−18.8	0.65

^*∗*^ *t*-test comparison, *p*  <  0.05 significant. LVIDd, left ventricle end diastolic diameter; MV, mitral valve.

**Table 4:** Correlation between duration of pericardial effusion and pretransplant cardiac markers.

Correlation with duration of effusion^*∗*^
	*R* value	*p* value
Ejection fraction	0.312	0.26
Shortening fraction	−0.355	0.19
Hemoglobin	−0.337	0.29
BSA	−0.137	0.66
LVIDd *z*-score	0.522	0.07^*∗*^
MV *E*/*A*	−0.198	0.52
Mitral lateral *E*/*E*′	0.271	0.40
Apical 4 Ch LV peak average strain	0.684	0.04^*∗*^

^*∗*^Statistically significant. Pearson correlation, 2 tail. LVIDd, left ventricle end diastolic diameter; MV, mitral valve.

## Data Availability

The data used to support the findings of this study are available from the corresponding author upon reasonable request.
